# Role of probiotics in managing various human diseases, from oral pathology to cancer and gastrointestinal diseases

**DOI:** 10.3389/fmicb.2023.1296447

**Published:** 2024-01-05

**Authors:** Oana-Alina Petrariu, Ilda Czobor Barbu, Adelina-Gabriela Niculescu, Marian Constantin, Georgiana Alexandra Grigore, Roxana-Elena Cristian, Grigore Mihaescu, Corneliu Ovidiu Vrancianu

**Affiliations:** ^1^Microbiology-Immunology Department, Faculty of Biology, University of Bucharest, Bucharest, Romania; ^2^The Research Institute of the University of Bucharest, Bucharest, Romania; ^3^Academy of Romanian Scientists, Bucharest, Romania; ^4^Department of Science and Engineering of Oxide Materials and Nanomaterials, Politehnica University of Bucharest, Bucharest, Romania; ^5^Institute of Biology of Romanian Academy, Bucharest, Romania; ^6^National Institute of Research and Development for Biological Sciences, Bucharest, Romania; ^7^Department of Biochemistry and Molecular Biology, Faculty of Biology, University of Bucharest, Bucharest, Romania

**Keywords:** microbiome therapeutics, dysbiosis, intestinal barrier, probiotics, gut microbiota

## Abstract

The imbalance of microbial composition and diversity in favor of pathogenic microorganisms combined with a loss of beneficial gut microbiota taxa results from factors such as age, diet, antimicrobial administration for different infections, other underlying medical conditions, etc. Probiotics are known for their capacity to improve health by stimulating the indigenous gut microbiota, enhancing host immunity resistance to infection, helping digestion, and carrying out various other functions. Concurrently, the metabolites produced by these microorganisms, termed postbiotics, which include compounds like bacteriocins, lactic acid, and hydrogen peroxide, contribute to inhibiting a wide range of pathogenic bacteria. This review presents an update on using probiotics in managing and treating various human diseases, including complications that may emerge during or after a COVID-19 infection.

## 1 Introduction

The gut microbiome represents an intricate ecosystem where microorganisms and their metabolic products engage with host cells, exerting an influence on various bodily functions (Ma et al., 2023). Overall health is closely linked to a “healthy” microbiome, characterized by various bacterial species residing in the gut (Hills et al., 2019; Shanahan et al., 2021). A multitude of environmental variables (e.g., patterns and methods of newborn feeding, delivery mode, dietary patterns, and antibiotic usage) all impact the overall patterns of microbial colonization in the gastrointestinal tract during early life (Beharry et al., 2023; Khan A. et al., 2023; Khan M. T. et al., 2023; Xiao and Zhao, 2023). The disruption of the early colonization process by a “healthy” microbiome has been linked to increased susceptibility to immune-mediated illnesses, such as allergies (Peroni et al., 2020; Parkin et al., 2021). Several factors, including the individual’s age, overall health, geographic location, antibiotic treatment dosage and duration, and specific antibiotics, influence the recovery of the gut microbiota following the antibiotic-induced disruption (FitzGerald et al., 2022). Antibiotic treatment and the resulting resistant bacteria can substantially impact the diversity and makeup of the gut’s bacterial microbiota. This disruption can potentially diminish or even eliminate specific microbial species, hence the networking within the bacterial community and with the host (Duan et al., 2022; Patangia et al., 2022). These changes stem from shifts in how intestinal epithelial cells produce mucin, cytokines, and antimicrobial peptides. Reports have pointed out that dietary strategies involving probiotics, prebiotics, omega-3 fatty acids, and the addition of butyrate (Thomas et al., 2021), as well as procedures like fecal microbiota transplantation (Nigam et al., 2022; Quaranta et al., 2022), have shown promise in counteracting the disruptive effects of antibiotic-induced gut dysbiosis and the subsequent harm to the gut barrier (Yadav et al., 2022).

Numerous intestinal bacteria, including *Lactobacillus* (Dempsey and Corr, 2022; Louis et al., 2022; Rastogi and Singh, 2022), *Bifidobacterium* (Sadeghpour Heravi and Hu, 2023), and *Enterococcus* (Im et al., 2023), contribute positively to gut microbiota stability. This imbalance in the microbiome, known as dysbiosis, allows potentially harmful bacteria like *Clostridium perfringens*, *Staphylococcus aureus*, or *Clostridioides difficile* to gain prominence (Ribeiro et al., 2020; Elias et al., 2023) and has also been associated with various health concerns such as obesity, malnutrition, inflammatory bowel disease, neurological disorders, and cancer (Horii et al., 2021).

Beneficial microorganisms and their mutually beneficial relationship with humans are pivotal in maintaining human health (Suman et al., 2022). A deep understanding of this complex interaction has paved the way for innovative personalized healthcare strategies. Probiotics, microorganisms with several health advantages when consumed in adequate quantities, have garnered substantial attention for their potential to prevent and treat a wide range of diseases (Bodke and Jogdand, 2022). Probiotics offer multifaceted benefits to human health, including antimicrobial (Fijan, 2023), anti-inflammatory (Cristofori et al., 2021; Aghamohammad et al., 2022), antioxidant (Rwubuzizi et al., 2023), and immunomodulatory properties (Aghamohammad et al., 2022), alleviating lactose intolerance (Ahn et al., 2023), addressing diarrheal diseases (Kora, 2022), aiding in ulcer treatment (Awasthi et al., 2022), stimulating immunity (Liu G. et al., 2022; Liu Y. et al., 2022), preserving food (Teneva and Denev, 2023), and potentially mitigating colon cancer risk (Singh et al., 2022). Consequently, harnessing the power of probiotics represents a straightforward, cost-effective, adaptable, and inherent approach to achieving improved outcomes in human health (Yadav et al., 2022).

This review presents an update on the utilization of probiotics in managing and treating various human diseases, including complications that may emerge during or after a COVID-19 infection.

## 2 General characterization of probiotics

**Figure 1:**
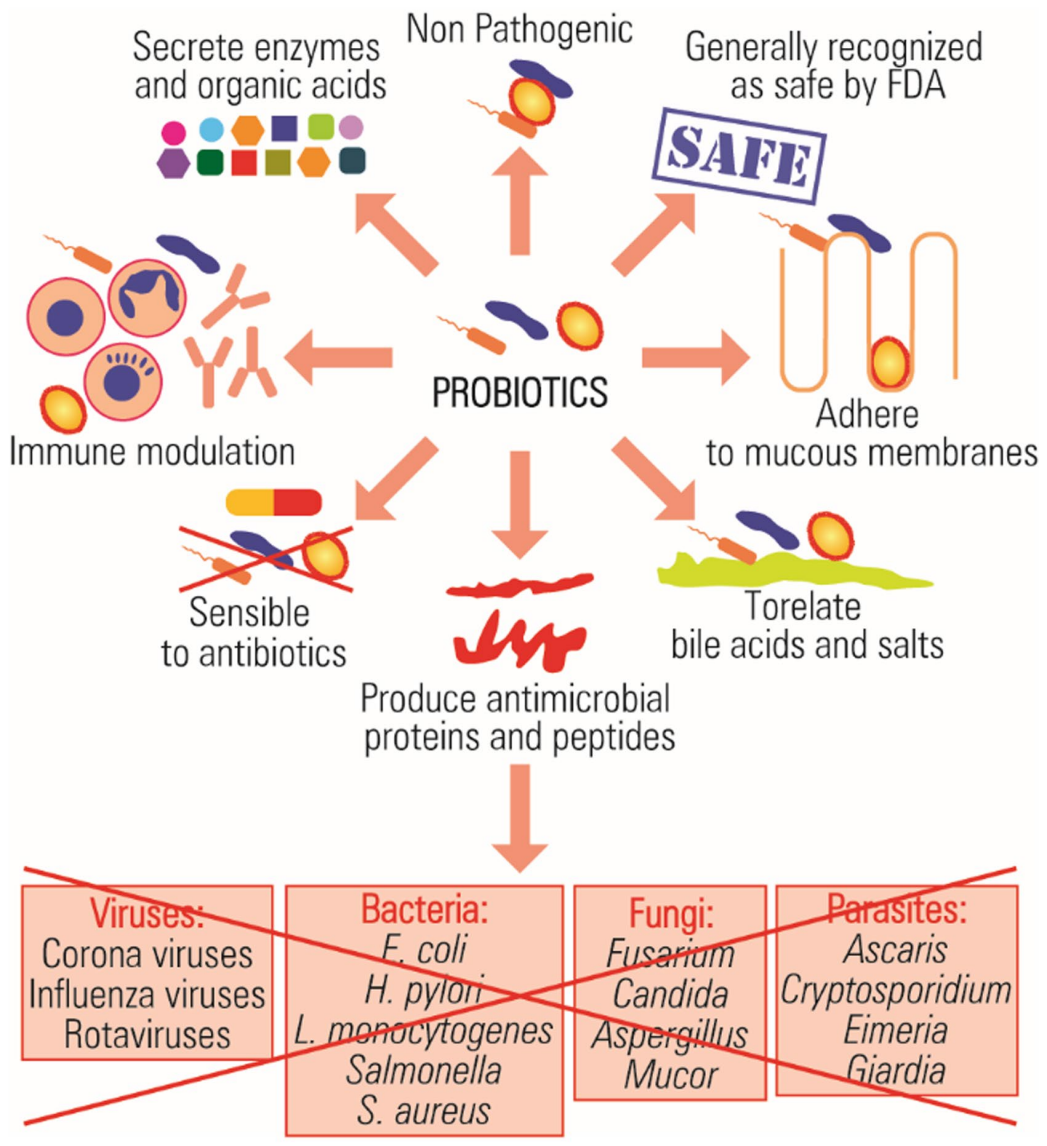
Mechanisms of the antimicrobial action of probiotics.

Probiotics are microorganisms that engage in a mutually beneficial relationship with the host organism, offering health advantages and performing crucial biological functions when administered in sufficient quantities (Suman et al., 2022). When choosing probiotic strains, specific criteria are employed to ensure compliance with safety and effectiveness standards (Wang et al., 2021). The World Health Organization (WHO) has established criteria for the *in vitro* assessment of probiotics, encompassing safety, effectiveness, functionality, and potential applications in technological and physiological contexts (FAO/WHO, 2002). Probiotic strains chosen for evaluation can be characterized by various vital properties, including their non-pathogenic nature, ability to withstand changes in the human gastrointestinal environment, capacity to adhere to and colonize the intestinal epithelium, antimicrobial properties, genetic and phenotypic stability, and immunomodulatory capabilities (Figure 1; de Melo Pereira et al., 2018; Roe et al., 2022).

Previous research has categorized probiotics into viable and active probiotics and viable inactive probiotics (Silva et al., 2020). The most consumed probiotics in human nutrition belong to genera such as *Lactobacillus*, *Bacillus*, *Bifidobacterium*, and *Saccharomyces*. Lactic acid bacteria (LAB) are considered the main probiotic bacteria that are used as viable cells, including homofermentative lactobacilli, which are represented by three main groups, including *L. acidophilus*, *L. salivarius*, and *L. rhamnosus* (Zielinska and Kolozyn-Krajewska, 2018). Probiotic lactic acid bacteria show significant promise as substitutes for antibiotics, serving as both preventive and curative treatments (Silva et al., 2020; van Zyl et al., 2020; Wang et al., 2022).

In addition, among producers of non-lactic acid are *B. cereus, E. coli Nissle 1917*, *Sporolactobacillus inulinus*, *Propionibacterium freudenreichii*, and *Saccharomyces cerevisiae* (Fijan, 2014). The applications of selected probiotics against pathogenic bacteria and their mechanisms of action are summarized in Figure 1. Notably, *Bacillus* species like *B. coagulans*, *B. subtilis*, *B. clausii*, and *B. licheniformis* have recently gained recognition as viable yet inactive probiotics, finding applications in human nutrition, treatment of intestinal and urinary issues, childhood diarrhea, and managing respiratory infections (Bu et al., 2022; Luise et al., 2022; Aysha Jebin and Suresh, 2023; Bahaddad et al., 2023).

## 3 Properties and effect mechanisms of probiotics

### 3.1 Effect mechanisms of probiotics

Probiotics serve a broad spectrum of health-related purposes, which include improving oral and intestinal health, preventing diarrhea, strengthening the immune system, lowering serum cholesterol levels, and exhibiting various beneficial effects such as antimicrobial, anti-biofilm, antioxidant, anti-inflammatory, and anti-diabetic properties. It’s important to note that these health-promoting qualities are contingent on the specific probiotic strain in use, and each exerts its effects through distinct mechanisms (Pique et al., 2019). Probiotics have several benefits: (i) reduce vulnerability to infections; (ii) reduce lactose intolerance, relieve allergic episodes and respiratory infections; (iii) reduce serum cholesterol and blood pressure; (iv) prevent the intestine from gastritis and diarrhea; (v) prevent urogenital and vaginal infections and (vi) reduce the chances of colon cancer (Reid et al., 2003).

Some probiotic strains have been found to produce a range of small molecules that can have specific localized effects. These molecules encompass acetylcholine, oxytocin, norepinephrine, dopamine, serotonin, tryptamine, and gamma-aminobutyric acid. Furthermore, research on rats has indicated that probiotics can influence adrenocorticotropic hormone levels and corticosterone levels (Liang et al., 2015).

**Figure 2:**
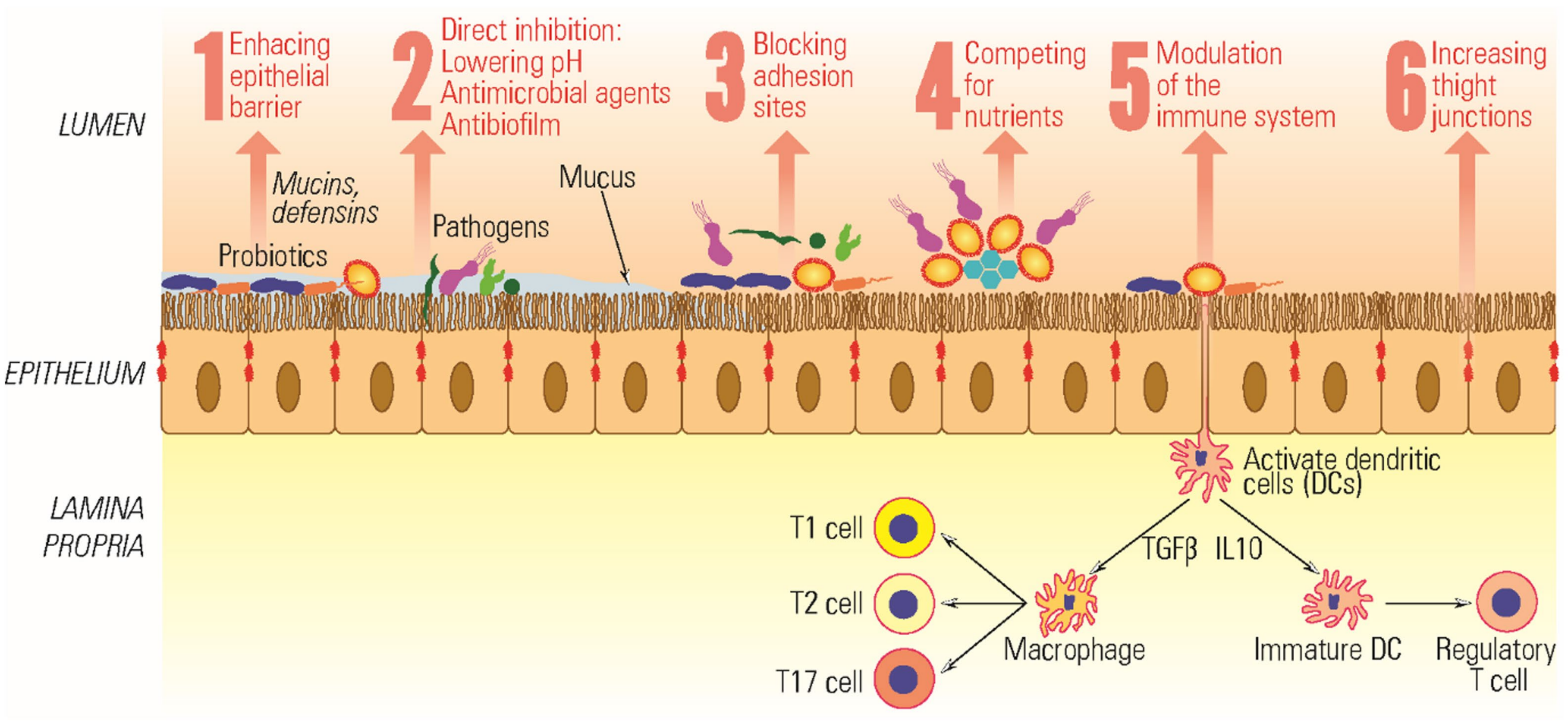
Mechanism of action of probiotics.

Before embarking on clinical trials, several *in vitro* tests are available to assess the effectiveness of probiotics. These include methods such as the agar spot test, agar well diffusion test, microdilution assays, antibiofilm assessments, 3D cell cultures, and the utilization of human tissues and animal models (Papadimitriou et al., 2015). Furthermore, probiotics have shown potential in aiding various medical conditions, including but not limited to constipation, diarrhea, polycystic ovary syndrome, ulcerative colitis, stress and anxiety, inflammatory bowel disease, breast cancer, and diabetes (Bodke and Jogdand, 2022). Probiotics also exert antimicrobial effects and modulate the mucosal immune system through several mechanisms summarized in Figure 2.

### 3.2 Anti-inflammatory properties

Probiotic bacteria have been found to significantly influence the immune system by releasing anti-inflammatory cytokines within the gut. They can potentially impact various types of immune cells, including dendritic cells, monocytes, natural killer (NK) cells, macrophages, lymphocytes, and epithelial cells. A key mechanism of action involves the activation of pattern recognition receptors (PRRs) found on both immune and non-immune cells (Ferlazzo et al., 2011). However, the precise molecular interactions between probiotics and the host organism still need to be fully understood and require further research. Specific strains of *Lactobacillus* sp. have been found to influence cytokine production, while *Bifidobacterium* sp. strains have been associated with promoting immune tolerance (Djaldetti and Bessler, 2017; Azad et al., 2018). Oral administration of probiotic strains like *L. plantarum*, *L. acidophilus*, *B. breve*, and *B. lactis* influence the release of proinflammatory cytokines through toll-like receptor signaling pathways (Yao et al., 2017; Asgari et al., 2018). These varying regulatory activities are associated with the structural characteristics of the probiotic strains, the range of mediators they release, and the different immune pathways they activate simultaneously (Hoffmann et al., 2014).

*Bifidobacterium* sp. and *Lactobacillus* sp. play significant roles in modulating various aspects of the immune system, including the humoral response, cell-mediated responses, and non-specific immunity. Probiotics such as these can enhance the secretion of immunoglobulin IgA, thus influencing humoral immunity and helping the body defend against invading pathogens. A similar effect was observed when individuals consumed yogurt containing *L. casei* and *L. acidophilus*, resulting in a substantial increase in IgA-producing plasma cells (Cristofori et al., 2021). Moreover, research conducted by Hasan and collaborators demonstrated the ability of heat-killed probiotic *Bacillus* sp. SJ-10 to act as a potent modulator of the innate immune response. This research was conducted in olive bream, underscoring the broad applicability of probiotics in enhancing the innate immune system’s capabilities (Hasan et al., 2019).

The commensal microbiota maintains the epithelial environment’s balance by stimulating the production of epithelial repair factors and regulating the immune response, safeguarding against epithelial damage. When administered orally, probiotics activate TLR signaling, producing cytokines that activate macrophages and influence intestinal epithelial cells (IEC) and immune cells within the lamina propria. This activation, in turn, stimulates regulatory T cells to release IL-10. Probiotics exhibit immunomodulatory effects that impact humoral immunity, cell-mediated immunity, and the non-specific immune response (Maldonado Galdeano et al., 2019). The regulation of proinflammatory cytokines by probiotics has been shown to decrease inflammation in the gingival area (Ma et al., 2023). Among the commonly used probiotic strains are various *Lactobacillus* and *Bifidobacterium* sp., including *L. acidophilus*, *L. casei*, *L. rhamnosus*, *L. reuteri*, *L. johnsonii*, *L. gasseri*, *B. longum*, *B. bifidum*, and *B. infantis*. Several studies have highlighted the significance of *L. fermentum* and *L. gasseri* in healthy individuals, as they are responsible for inhibiting periodontal pathogens such as *Porphyromonas gingivalis*, *Prevotella intermedia*, and *Aggregatibacter actinomycetemcomitans*. This inhibition is achieved through various mechanisms, including the production of hydrogen peroxide, antibacterial substances like bacteriocins, and the generation of inorganic acids. By doing so, these probiotic strains contribute to maintaining the dynamic balance of normal microbiota, ultimately restoring homeostasis (Jansen et al., 2021).

### 3.3 Antimicrobial properties

Probiotics present a promising approach to combat the rise of antibiotic-resistant bacteria (Knipe et al., 2021). Probiotics employ several key antimicrobial mechanisms to achieve this goal, including competitive exclusion, intestinal barrier function improvement by enhancing mucin and tight junction protein expression, antimicrobial molecule secretion, and immune system regulation (Silva et al., 2020).

The antimicrobial activity of probiotics relies significantly on their ability to produce antimicrobial peptides, which play a central role in competitively excluding pathogens. *Lactobacillus* strains, in particular, have demonstrated significant antimicrobial activity against various pathogens, including *Klebsiella* sp., *Clostridium difficile*, *Shigella* sp., *E. coli*, *P. aeruginosa*, *S. mutans*, and *S. aureus* (Prabhurajeshwar and Chandrakanth, 2019). They employ multiple strategies to outcompete and inhibit these pathogenic bacteria, including producing lactic acid, bacteriocin, and hydrogen peroxide, inhibiting the adhesion of pathogenic bacteria to the mucosa and improving the immune response (Plaza-Diaz et al., 2017).

The intestinal barrier serves as a critical line of defense to maintain the homeostasis of the intestine. It accomplishes this by performing various mechanical, chemical, immune, and microbial barrier functions (Vancamelbeke and Vermeire, 2017). Probiotics can improve and reinforce the integrity of the gut barrier through various mechanisms, including upregulating genes and protein expression involved in tight junction signaling. Probiotics can also regulate the apoptosis and proliferation of intestinal epithelial cells, contributing to barrier repair and maintenance (Gou et al., 2022). For instance, *L. acidophilus* can induce rapid and strain-specific enhancement of intestinal epithelial tight junction barrier function. This effect is mediated through TLR complexes, specifically TLR-2/TLR-1 and TLR-2/TLR-6. Strengthening tight junctions helps protect against intestinal inflammation (Al-Sadi et al., 2021). Probiotics can induce mucin expression and promote mucus secretion by goblet cells. Studies have demonstrated that treating mucus-secreting colon epithelial cells with supernatants from a probiotic-rich yogurt mixture can elevate mucin protein expression, including MUC2, a vital constituent of the protective mucus layer. CDX2, a regulator of MUC2 expression, is also influenced by probiotics (Chang et al., 2021).

**Figure 3:**
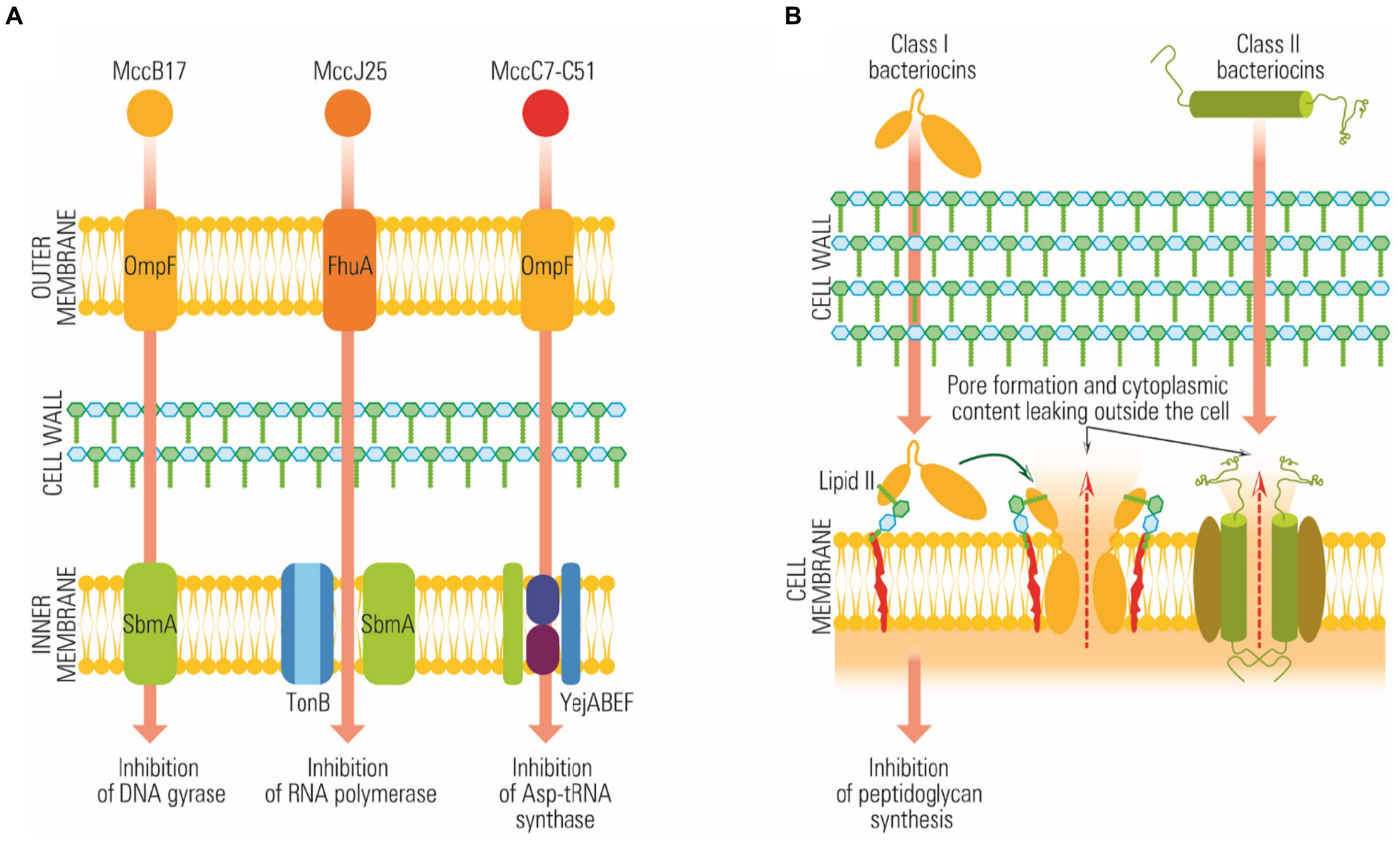
Schematically representation of bacteriocins mode of action. **(A)** The action of bacteriocins on Gram-negative targets, without the formation of pores. **(B)** The action of bacteriocins on Gram-positive targets, with the formation of membrane pores.

The primary method by which *Lactobacillus* strains exhibit antimicrobial activity is by releasing substances called bacteriocins (La Storia et al., 2020). Bacteriocins are antimicrobial peptides that combat many bacteria, including Gram-positive and Gram-negative species. Interestingly, the bacteria that produce these bacteriocins have developed specific mechanisms to protect themselves from the action of their antimicrobial peptides (Perez-Ramos et al., 2021). Both *Bifidobacterium* and *Lactobacillu*s strains are known to be producers of bacteriocins. These antimicrobial peptides function through various means, which include inhibition of lipid II, a critical component of bacterial cell membranes, prevention of peptidoglycan synthesis, and pore formation. The pore formation process often involves a receptor known as the mannose-phosphotransferase system, as depicted in Figure 3 and Martinez et al. (2013).

### 3.4 Antioxidant properties

The antioxidant properties of probiotics can be attributed to several actions. Probiotics can chelate metal ions, such as iron, thereby reducing their availability for catalyzing the production of harmful ROS. Certain probiotic strains can synthesize antioxidant metabolites, including vitamins like C and E, glutathione, and various ROS-scavenging enzymes, which help mitigate oxidative stress. Probiotics can stimulate the host’s production of antioxidants or enhance dietary antioxidants’ absorption, bolstering the body’s overall antioxidant capacity. Probiotics can influence oxidative stress and inflammation by modulating signaling pathways, ultimately regulating ROS production. Some probiotic strains can downregulate enzymes responsible for ROS generation, such as NADPH oxidase, leading to decreased ROS levels and reduced oxidative stress. Probiotics also play a role in shaping the composition of the intestinal microbiota, indirectly impacting gut health and potentially contributing to the reduction of oxidative stress (Wang et al., 2017).

Nuclear factor erythroid 2–related factor 2 (Nrf2) is a component belonging to the cap’n’collar transcription factor family and comprises seven NEH domains. In response to OS, Nrf2 plays a critical role in the ubiquitin-dependent signaling pathway (Seminotti et al., 2021). When ROS levels are elevated, Nrf2 disengages from its constant inhibitor, Keap1, migrates to the nucleus, and forms a complex with antioxidant response element (ARE) sequences, thereby initiating the transcription of genes involved in antioxidation, such as NQO1, GST, HMOX1, GCL, and GSH. Substantial research suggests that the activation of Nrf2 can inhibit oxidative stress and inflammation, thus potentially averting conditions like UC (Trivedi et al., 2016).

Probiotics are thought to activate the Nrf2 system in the host, representing a crucial mechanism underlying their antioxidant properties. Several *in vitro* studies have illuminated the role of Nrf2 pathway activation in mediating the antioxidant effects of specific probiotic strains such as *B. infantis*, *C. butyricum*, and *L. casei Shirota* in the context of intestinal injury (Ehrlich et al., 2020; Dou et al., 2021). TLRs activation has also been shown to stimulate Nrf2-ARE signaling and heme oxygenase-1 (HO-1) both *in vivo* and *in vitro* (Nadeem et al., 2016). Moreover, several studies have documented that the activation of TLR-Nrf2 signaling by *Lactobacillus* sp. could account for their antioxidant advantages within the gastrointestinal systems of piglets (Yang et al., 2022).

Oxidative stress arises from either the cumulative production of ROS or the inadequate scavenging activity of the antioxidant system, resulting in a disturbance in the body’s redox balance (Pizzino et al., 2017). Whether taken alone or alongside food, probiotic consumption has demonstrated the capacity to enhance antioxidant activity, thereby mitigating tissue damage caused by oxidative processes (Wang et al., 2017). Among the various antioxidant activities exhibited by probiotic strains such as *Lactobacillus* sp., *Bifidobacterium* sp., and *Propionibacterium* sp., it has been observed that *P. freudenreichii* displays the highest antioxidant activity (Amaretti et al., 2013). These probiotics release potent antioxidant compounds, including vitamin E, vitamin C, glutathione, beta-carotene, superoxide dismutase (SOD), polysaccharides, prototypical coenzyme I (NADH), and certain unidentified substances, all of which contribute to the promotion of gut health (Hemarajata and Versalovic, 2013). Additionally, probiotics can reduce oxidative stress by inhibiting cytokine production and decreasing levels of interleukin 1 and tumor necrosis factor-alpha while simultaneously increasing glutathione (GSH) levels (Pourrajab et al., 2022).

Various *Lactobacillus* strains, including *L. johnsonii*, *L. reuteri*, *L. brevis* and *L. fermentans* have been observed to activate the NF-κB pathway. In studies conducted with rats, this activation has been associated with reduced OS and inflammation in the intestinal tract (Pan et al., 2016; Wu et al., 2018). Interestingly, genetically engineered *Lactobacillus* strains expressing superoxide dismutase (SOD) have also shown similar positive effects. Additionally, *Bifidobacterium* sp. has been found to downregulate ROS production and inhibit the NF-κB pathway, contributing to the modulation of the intestinal immune system and protecting the intestinal epithelium (Yao et al., 2021). The interaction between SIRT1 and Nrf2/ARE is a critical component of the antioxidant defense system. SIRT1 facilitates the nuclear translocation of Nrf2, consequently elevating the expression of antioxidant proteins and phase II detoxification enzymes. In rats with aging-induced colitis, treatment with *Lactobacillus* C29 led to a decrease in plasma levels of ROS, malondialdehyde (MDA), and C-reactive protein while also increasing SIRT1 expression (Kim et al., 2019). Moreover, the mitigation of high-fat-diet-induced ulcerative colitis (UC) by administering *B. longum* and *L. plantarum* is linked to the activation of SIRT1. Additionally, studies have shown that activated SIRT2 can deacetylate forkhead box proteins (FOXO1a and FOXO3a), thereby augmenting the expression of antioxidant enzymes under the regulation of FoxO (Kitada et al., 2019). The activation of manganese superoxide dismutase (Mn-SOD)/SOD2 by *B. longum* and *L. acidophilus* to reduce cellular ROS levels, mediated by SIRT2, has been reported (Guo et al., 2017). Additionally, LGG has been shown to prevent H2O2-induced disruption of tight junctions in the human intestinal epithelium, possibly mediated through the ERK1/2 pathway (Zheng et al., 2022). Heat-killed and active *L. brevis* have effectively ameliorated subtotal duodenal and colonic injury caused by dextran sulfate sodium by mitigating oxidative stress and inflammation through a p38-MAPK-mediated pathway (Jiang et al., 2018).

In summary, probiotics exhibit antioxidant properties through several mechanisms, including scavenging free radicals, chelation of metal ions, regulation of antioxidant enzyme expression, and modulation of the gut microbiota. These effects are mediated at the molecular level by the influence of probiotics on various signaling pathways, such as Nrf-2, NF-κB, MAPK, and SIRTs, allowing them to exert their beneficial antioxidant effects.

## 4 Next-generation and genetically modified probiotics

The rapid evolution of high-throughput sequencing technology and the expanding field of bioinformatics have significantly deepened our understanding of the intricate relationship between the human microbiome (Zhu et al., 2010), particularly in the gut, and a wide range of human diseases. This progress has led to a breakthrough in isolating and identifying numerous gut microorganisms that were previously challenging to cultivate in laboratory settings (Browne et al., 2016; Brown et al., 2022). Many strains with promising health benefits have emerged among these newly discovered microbes. These potential strains are now prime candidates for developing next-generation probiotics (NGPs). They promise to become active biological agents in clinical contexts, offering targeted treatments for specific diseases. This dynamic development underscores the evolving landscape of microbiome research and its potential to revolutionize personalized healthcare approaches (Khan A. et al., 2023; Khan M. T. et al., 2023).

NGPs are specific strains of commensal microbes commonly found within the GIT, influencing the host’s natural defenses against gastrointestinal pathogens. NGPs comprise gut bacteria with specific nutritional requirements and oxygen sensitivity, including genera such as *Bacteroides*, *Clostridium*, *Faecalibacterium*, and *Akkermansia*, as well as genetically engineered (GE) strains (Zhang H. et al., 2022). However, working with NGPs presents unique challenges. Their demanding nutritional needs and sensitivity to oxygen make mass production and maintaining their viability during processing and formulation quite challenging. Moreover, NGPs are not universally suitable or safe for all consumers. Unlike conventional probiotics, their application requires careful consideration of target consumers and specific circumstances (O’Toole et al., 2017).

In the context of gut microbiota, *Bacteroides* spp., such as *B. thetaiotamicron*, play a crucial role as essential members, possessing a remarkable ability to metabolize complex polysaccharides, allowing them to interact with intestinal cells. Moreover, they can modulate host gene expression by inducing immunotolerance in dendritic cells (Durant et al., 2020). Another significant player in the gut microbiota is *B. fragilis*, which stands out due to its zwitterionic polysaccharide that enables it to stimulate the host’s immune system (Chang et al., 2016). *C. butyricum*, a Gram-positive, spore-forming, butyrate-producing anaerobe commonly found in human and animal intestines, is known for the beneficial effects of butyrate production on the host. However, it’s important to note that *C. butyricum* supplementation in neonates can have adverse effects, including the induction of necrotizing enterocolitis and botulism type E (Cassir et al., 2016). *Faecalibacterium prausnitzii*, a non-spore-forming, Gram-negative, butyrate-producing anaerobe, is another key member of the gut microbiota. Its extremely oxygen-sensitive nature has made working within laboratory settings challenging (Lopez-Siles et al., 2017). Consequently, many experiments involving *F. prausnitzii* utilize culture supernatants (SN) rather than live cells to circumvent viability and stability issues. This species is particularly interesting due to its immunomodulatory activities and role in butyrate synthesis, contributing to potential health benefits. Moreover, *F. prausnitzii* has demonstrated the ability to prevent colitis induced by various compounds, including dinitrobenzene sulfonic acid (DNBS), trinitrobenzene sulfonic acid (TNBS), or dextran sulfate sodium (DSS) (Martin et al., 2014). Furthermore, live cells of *F. prausnitzii* have shown promise in reducing the incidence of diarrhea and related mortality in dairy calves and promoting increased body weight (Foditsch et al., 2015).

## 5 Probiotics as therapeutic tools

### 5.1 Probiotics in gastrointestinal diseases

#### 5.1.1 Probiotics in diarrhea and constipation

Probiotics treat acute diarrhea as they activate immunological signaling pathways, produce anti-pathogenic factors, and cause the host to secrete them to combat enteric infections. A study conducted by Rashad Hameed and Abdul Sattar Salman aimed to evaluate the effects of 11 isolates of probiotics bacteria (including three *L. plantarum*, one *L. gasseri*, two *L. fermentum*, three *L. acidophilus*, and two *L. garvieae* isolates) against diarrheal causative bacteria. The results showed that probiotic isolates had antibacterial and co-aggregative effects against diarrhea-causative bacteria with an inhibition diameter of 17–49 mm for different *Lactobacillus* spp. and *Lactococcus* spp. isolates (Rashad Hameed and Abdul Sattar Salman, 2023). Higuchi and collaborators led a PROSPERO meta-analysis (CRD 42023405559), including 14 randomized controlled studies to investigate the efficacy of probiotics utilized in Japan in treating acute gastroenteritis in children under 18 years. They evaluated several aspects of data on diarrhea duration, number of hospitalizations, length of hospital stay, and adverse effects. The results showed that diarrhea lasting longer than 48 h and duration of hospitalization were significantly lower in the probiotic group (Higuchi et al., 2023).

A recent meta-analysis delved into the efficacy of probiotics in managing acute diarrhea, revealing notable outcomes in both children and adults. The analysis highlighted a significant 26% reduction in the duration of acute diarrhea in children following probiotic intervention. However, intriguingly, no discernible impact on the risk of hospitalization was observed in this group (Huang et al., 2021). Zheng and collaborators conducted two randomized sub-trials in subjects diagnosed with functional constipation or diarrhea to determine the efficacy of four-week probiotic supplementation on gastrointestinal health. In each sub-trial, 70 eligible adults were randomized to receive a multi-strain probiotic combination or a placebo. The authors assessed several aspects, like gastrointestinal symptoms, defecation habits, stool characteristics, blood and fecal biochemistry markers, anthropometrics measures, stress-associated responses, and intestinal flora changes. The results showed that 4 weeks of probiotic supplementation reduced overall gastrointestinal symptom scores in participants with functional constipation. For participants with functional diarrhea, probiotics consumption markedly reduced overall gastrointestinal symptom scores and decreased stool frequency thrice weekly (Zheng et al., 2023a).

A meta-analysis involving six randomized controlled trials and 444 geriatric patients revealed that when compared to a placebo, probiotics led to increased stool frequency and a notable impact on ameliorating symptoms associated with constipation (Deng et al., 2023). Recently, Patch and collaborators conducted a double-blinded randomized controlled trial registered in 2021 at the Australian New Zealand Clinical Trials Registry (ACTRN12621001441808p). This study aimed to use a proprietary strain of deactivated *B. subtilis* to treat self-reported functional gastrointestinal disorders in 67 participants. The reports revealed that constipation and diarrhea in the experimental group were improved by 33%/26% compared to placebo (15%/22%). Further analysis revealed that the intervention group’s clusters for constipation, indigestion, and dyspepsia were significantly improved compared to the placebo (Patch et al., 2023). Dong and collaborators investigated seven effectiveness indicators and the incidence of adverse reactions in the treatment of constipation (treatment success rate, defecation frequency, frequency of abdominal pain, stool consistency, frequency of defecation pain, frequency of fecal incontinence, and recurrence rate) in nine meta-analyses and systematic reviews. According to the results, this study revealed that the intake of probiotics in children with functional constipation significantly improved treatment success rate and defecation frequency while decreasing the recurrence rate of constipation. The intake of probiotics did not increase the incidence of adverse reactions and demonstrated good safety (Dong et al., 2023).

Another systematic review (PROSPERO ID: CRD42020195869) conducted by Liu and collaborators included seventeen randomized controlled trials involving 1,504 children diagnosed with functional constipation. This study evaluated the efficacy and safety of probiotics and synbiotics in treating childhood constipation and the impact on treatment success, defecation frequency, stool consistency, painful defecation, fecal incontinence, and adverse events in treating childhood functional constipation. Compared to placebo, probiotics significantly improved defecation frequency and fecal incontinence. However, it did not significantly improve treatment success, painful defecation, and abdominal pain (Liu J. et al., 2023).

Radiotherapy and chemotherapy are commonly used therapeutic approaches for cancer patients. However, they often lead to diarrhea, significantly impacting the patient’s quality of life and adversely affecting the overall treatment outcomes. However, when administered alongside radiotherapy and chemotherapy, *L. acidophilus* has been shown to reduce the duration of diarrhea in pediatric patients and even inhibit rotavirus infection (Lee et al., 2015). LGG has demonstrated its effectiveness in managing various types of diarrhea, including infectious diarrhea, ulcerative colitis, antibiotic-associated diarrhea, and rotavirus-induced diarrhea. It achieves this by stimulating the production of mucosal immunoglobulin A (IgA) and secretory IgA (sIgA) (Li et al., 2019). The prophylactic and therapeutic oral administration of *B. bifidum G9-1* has ameliorated rotavirus-induced gastroenteritis. It achieves this by producing mucosal protective peptides that effectively manage rotavirus-induced gastroenteritis (Kawahara et al., 2017).

#### 5.1.2 Probiotics in *Helicobacter pylori* infection

*Helicobacter pylori*, often known as *H. pylori*, is a widely recognized pathogen that infects over 50% of the global population. This is a pathogenic microorganism that can cause health problems since it has several features that make it harmful and virulence genes that are associated with gastrointestinal issues. Additionally, it might potentially lead to immune thrombocytopenic purpura and elevate the risk of cardiovascular disease as acute coronary syndrome and also neurological effects (Tsay and Hsu, 2018). The declining efficacy of current therapeutic regimens in eradicating *H. pylori* poses a substantial challenge for the medical community. Although several therapeutic methods have been utilized, the addition of probiotics as a supplementary therapy has shown encouraging results (Bai et al., 2022).

In the treatment of *H. pylori* infection, the conventional triple therapy includes the use of clarithromycin. However, in areas where there is a high or uncertain level of antibiotic resistance, it is suggested to use either bismuth quadruple therapy or non-bismuth medicines (Malfertheiner et al., 2022). Nevertheless, research on probiotics is expanding as eradication rates continue to decline. Based on our understanding, the use of a *Lactobacillus reuteri* probiotic as an additional medication in managing individuals infected with *H. pylori* has been shown to be advantageous, as demonstrated by prospective, randomized, double-blind, placebo-controlled trials (Emara et al., 2014; Ismail et al., 2022, 2023).

The benefit of probiotics has been assessed in meta-analysis studies, which included 34 eligible randomized controlled trials. These trials mostly focused on triple therapy and showed enhanced eradication rates and decreased occurrence of adverse effects when it was used in combination with different probiotics (Wang et al., 2023a). There are many alternative eradication therapies based on probiotics as adjuvant agents (Penumetcha et al., 2021), such as the combination of multiple antibiotic-resistant lactic acid bacteria preparations and Vonoprazan (Kakiuchi et al., 2020), capsules containing four probiotics (Telaku et al., 2022) and combined mixtures of medicinal plants extracts with different probiotic strains (Hasna et al., 2023).

#### 5.1.3 Probiotics in inflammatory bowel disease

The gastrointestinal tract protects the body from toxic and infectious substances. However, factors like stress, unhealthy diets, dysbiosis, and antibiotic treatment for different pathogens can compromise the intestinal microbiota and barrier function, leading to increased permeability. This can cause diseases like inflammatory bowel disease and irritable bowel syndrome, as well as extra-intestinal conditions like heart disease, obesity, and type 1 diabetes (Aleman et al., 2023). Leaky gut syndrome is a medical disorder characterized by an abnormal increase in the permeability of the human intestine’s epithelium, which may allow harmful microorganisms, toxins, or food particles to enter the bloodstream and potentially impact various bodily systems, including the hormonal, immune, nervous, respiratory, and reproductive systems (Obrenovich, 2018; Aleman et al., 2023). The intestinal microbiota plays a crucial role in preserving the integrity and balance of the intestinal epithelium. The multi-strain probiotics are employed to modify the composition of gut microbiota and are recognized for enhancing the integrity of the intestinal barrier, hence boosting its protective effectiveness (Chaiyasut et al., 2022; Zheng et al., 2023b).

Inflammatory bowel disease (IBD) is a condition with an unknown origin, characterized by an exacerbated immune response in the body against its own gastrointestinal (GI) microflora, primarily affecting the colon and duodenum. IBD is typically classified into two main types: Crohn’s disease (CD) and ulcerative colitis (UC). While previous understanding pointed to adaptive immune responses as the main trigger for these conditions, recent research has highlighted the significant role of the innate immune system (So et al., 2023). The innate immune response can disturb the equilibrium between the human gut’s beneficial microbiome and commensal microflora. This disruption, known as dysbiosis, can lead to an intensified inflammatory response, thereby impacting the development of inflammatory bowel disease (IBD) (Roy and Dhaneshwar, 2023).

Recent research, encompassing both *in vivo* and *in vitro* studies, has provided substantial evidence that consuming probiotics in sufficient quantities is a potent defense against pathogenic and opportunistic microorganisms. Probiotics can restore the disrupted normal flora of the gastrointestinal tract, which can often occur due to dietary changes, surgical interventions, and antibiotic use in treating intestinal disorders (Das et al., 2022). Yakovenko and collaborators conducted a randomized clinical trial aiming to evaluate the effectiveness of a probiotic drug containing *B. longum* BB-46 and *Enterococcus faecium* ENCfa-68. This study included 62 patients diagnosed with post-infectious irritable bowel syndrome. The results affirmed that the probiotic product enhanced clinical symptoms of the disease, reinstated the natural balance of the intestinal microbiota, and alleviated inflammation in the intestinal mucosa. In most patients receiving probiotics, the remission of the disease was achieved at the end of the course of treatment and persisted even 6 months after its termination (Yakovenko et al., 2022). A clinical trial conceptualized by Bamola and collaborators assessed the impact of *B. clausii* UBBC-07 probiotic strain on gut microbiota and cytokines in IBD patients. The authors concluded that the probiotic strain modulated the gut microbiota and cytokine secretion, decreasing the IBD symptoms (Bamola et al., 2022). More recently, 24 patients with ulcerative colitis were included in a randomized clinical trial and administered probiotics containing nine *Lactobacillus* and five *Bifidobacterium* species. Probiotic therapy in ulcerative colitis patients has significantly improved the quality of life and systemic, social, and emotional scores (Rayyan et al., 2023).

### 5.2 Probiotics in cardiovascular and cerebrovascular diseases

Probiotics represent a promising avenue in the search for safe and natural alternatives to manage hypercholesterolemia and reduce the risk of cardiovascular disease (Chang et al., 2019). Widely distributed in human and animal body sites, including the intestines, mouth, respiratory tract, and reproductive tract, probiotic strains like *Bacteroides* are gaining attention for their contribution to cardiovascular health improvement (You et al., 2023). He and collaborators isolated 66 *Bacteroides* strains from healthy adult fecal samples and compared their ability to assimilate cholesterol. *In vitro* assessments of bile salt hydrolase activity revealed favorable probiotic characteristics in these strains, including a high survival rate during simulated gastrointestinal digestion, excellent adhesion capabilities, susceptibility to antibiotics, and the absence of hemolysis or virulence genes. Furthermore, the strains exhibited notable bile salt deconjugation activities associated with regulating cholesterol metabolism. The strains also displayed an upregulation of the BT_416 gene, which is linked to cholesterol. This provides valuable insights into a potential molecular mechanism underlying their cholesterol-reducing activity, suggesting a pathway through which these strains may contribute to improving cardiovascular health (He et al., 2023). Ruiz-Tovar and collaborators conducted a prospective non-randomized study to investigate the effect of *L. kefiri* and a hypocaloric diet on cardiovascular risk markers. The addition of *L. kefiri* to a low-calorie diet increased weight loss and further improved the glycemic and lipid profile and also caused a further improvement in obesity-associated dysbiosis, mainly by increasing the muconutritive and regulatory (e.g., *Bifidobacterium* spp., *Akkermansia muciniphila*) microbiome, and decreasing the *Firmicutes*/*Bacteroidetes* ratio (Ruiz-Tovar et al., 2023). In addition, recent studies demonstrated that chronic consumption of probiotics, oats, soymilk, and apples may decrease diastolic blood pressure, triglycerides, total cholesterol, and insulin levels and significantly increase high-density lipoprotein cholesterol, beneficially affecting cardiometabolic health (Naseri et al., 2022; Pavlidou et al., 2022; Hasanpour et al., 2023; Pushpass et al., 2023; Zarezadeh et al., 2023). A cross-sectional study involving a group of 190 schoolchildren from public schools in Brazil aimed to evaluate the fecal abundance of *Bifidobacterium* spp. and their relationship with food consumption and anthropometric characteristics. The results revealed that a high abundance of *Bifidobacterium* spp. is associated with a low prevalence of hyperglycemia and cardiovascular risk markers (Menezes et al., 2023). Two meta-analyses consisting of 16 randomized controlled trials investigating the role of several probiotics (*L. acidophilus*, *B. bifidum*, *L. reuteri*, *L. fermentum*, and *L. rhamnosus*) in treating coronary artery disease and lowering blood pressure, blood lipid, and blood glucose was conducted recently. These studies showed that adding probiotics to conventional medications for coronary artery disease reduced the levels of low-density lipoprotein cholesterol, fasting glucose, and hypersensitive C-reactive protein and increased the levels of high-density lipoprotein cholesterol and nitric oxide (Lei et al., 2023; Liu L. et al., 2023). Similarly, a retrospective cohort study of 4,837 participants aged 65 years or older found that subjects who used probiotics experienced a reduced risk of all-cause mortality by nearly 41% and cardiovascular mortality by 52% (Shen et al., 2023).

An ongoing open-label, randomized, controlled clinical trial (Chinese Clinical Trials Registry ChiCTR2000038797) involving 2,594 adult patients diagnosed with acute myocardial infarction aims to assess the effects of probiotics on in-hospital mortality and the incidence of major adverse cardiovascular events. Patients will receive a bifidobacteria triple viable capsule (*B. longum*, *L. acidophilus*, and *E. faecalis*) twice a day, along with standard treatment, for a maximum of 30 days or standard treatment strategy without the bifidobacterium triple live capsule. This clinical trial will provide evidence for probiotics as a complementary treatment for acute myocardial infarction (Chen et al., 2023).

In the case of stroke, probiotics can ameliorate neurological deficits, decreasing cerebral volume loss and inhibiting neuronal apoptosis by sustaining intestinal barrier function, such as increasing tight junctions, decreasing intestinal injury, and modulating gut microbiota (Wu and Chiou, 2021; Savigamin et al., 2022). A recent meta-analysis including 26 randomized controlled trials (2,216 patients) was conducted to explore the efficacy and safety of probiotics in stroke patients. The results showed a significantly lower incidence of gastrointestinal complications, a lower incidence of infection, a shorter length of hospital stay, and a lower dysbacteriosis rate in the probiotics group, suggesting that probiotics significantly improve the status of stroke patients (Chen et al., 2022).

### 5.3 Probiotics in oral diseases

One of the primary benefits of probiotics in the oral cavity is their ability to reduce inflammation. Probiotics play a role in preserving the health of gums and teeth by actively countering harmful microorganisms in the mouth. As a form of natural medicine, probiotics are generally considered safe and are not expected to yield adverse effects. Notably, *L. acidophilus* and *B. lactis* have gained recognition for their effectiveness in combating fungal infections in the oral environment (Lesan et al., 2017).

Oral probiotics should adhere to specific criteria to be effective without causing adverse effects. They should not ferment sugars, as this fermentation can decrease pH and potentially promote dental caries. Additionally, they should be capable of adhering to and colonizing all oral cavity tissues (Saiz et al., 2021). The oral cavity hosts a diverse community of bacteria belonging to several phyla, including *Firmicutes*, *Bacteroidetes*, *Proteobacteria*, *Actinobacteria, Spirochaetes*, and *Fusobacteria*, as revealed by research (Hernandez et al., 2022). These bacteria are typically normal commensals in healthy individuals, with *Candida* sp. among the most commonly observed organisms (Baumgardner, 2019).

The most common strains being a pillar in the probiotics development are *Lactobacillus* and *Bifidobacterium* sp. Notable strains within the *Lactobacillus* sp. include *L. acidophilus*, *L. johnsonii*, *L. rhamnosus*, *L. casei*, *L. reuteri*, and *L. gasseri*. These probiotic strains are associated with beneficial effects on oral health. They achieve this by engaging with toll-like receptor (TLR) signaling, which contributes to epithelial homeostasis by producing repair factors and immune regulation. This mechanism is crucial for protecting against epithelial injury. Furthermore, these probiotics play a role in regulating the release of inflammatory cytokines such as IL-1β and TNF-α, effectively reducing gingival inflammation (Ma et al., 2023).

#### 5.3.1 Probiotics in the prevention of dental caries progression

Probiotics play a significant role in maintaining and improving overall oral health. One approach involves using *Bifidobacterium* sp., including *B. dentium*, *B. breve*, *B. scardovii*, and *B. longum*, that are particularly effective in preventing the progression of deep dental caries (Abikshyeet et al., 2022). Another noteworthy probiotic, *L. lactis*, has been found to reduce the colonization of various oral bacteria, including *S. oralis*, *Veillonella dispar*, *Actinomyces naeslundii*, and the cariogenic *S. sobrinus* (Guo et al., 2023).

The research on *L. rhamnosus* has yielded promising findings regarding its positive impact on dental health. Consumption of milk containing *L. rhamnosus* has been associated with a beneficial effect in reducing the occurrence of dental caries and inhibiting the colonization of caries-causing streptococcal pathogens (Abikshyeet et al., 2022). Even after discontinuing yogurt consumption, a lasting reduction in caries incidence in children was observed. *L. rhamnosus* was also detected in saliva for an additional 2 weeks, indicating its potential for sustained oral health benefits. Additionally, short-term consumption of cheese containing *L. rhamnosus* significantly reduced caries-associated microbes, with a notable decrease in *S. mutans* (Amargianitakis et al., 2021; Homayouni Rad et al., 2023). Two research groups revealed that, when consumed daily, probiotic yogurt containing *Bifidobacterium* and *Lactobacillus* sp. has effectively reduced resistant streptococci associated with dental caries (Srivastava et al., 2020; Zare Javid et al., 2020). These findings underscore the potential of probiotics, particularly *L. rhamnosus* and *Bifidobacterium* strains, in promoting oral health and preventing dental caries.

Recent research has highlighted the potential of *L. paracasei* and *L. plantarum* in preventing dental caries by regulating the microbiota within dental plaques and inhibiting the *S. mutans* biofilms (Guo et al., 2023; Zeng et al., 2023). Furthermore, a systematic review led by Meng and collaborators, encompassing 17 randomized controlled trials, aimed to assess the clinical effectiveness of probiotics in preventing dental caries in preschool children. They concluded that probiotics can potentially reduce high levels of *S. mutans* in saliva. However, it was noted that probiotics did not significantly impact the levels of *Lactobacillus* sp. in both saliva and dental plaque (Meng et al., 2023). Pallavi and colleagues reported that *Enterobacter cloacae* strains isolated from yogurt exhibit probiotic properties. These strains can inhibit the cariogenic *S. mutans* bacterium by producing organic acids (Pallavi et al., 2023).

#### 5.3.2 Probiotics in the prevention of gingival inflammation

*Bifidobacterium* strains, including *B. dentium*, *B. breve*, *B. scardovii*, and *B. longum*, have been found to play a crucial role in inhibiting the adhesion of pathogens to oral sites, reducing plaque formation and gingival bleeding (Alkaya et al., 2017). One of the primary mechanisms through which these probiotic strains inhibit the aggregation of oral pathogenic microorganisms on tooth surfaces, thereby hindering the formation of biofilms, involves the disruption of surface adhesive proteins. Furthermore, *L. salivarius* has promising potential in combating subgingival microbiota (Nedzi-Gora et al., 2020).

Recent studies have spotlighted *L. reuteri* due to its possession of the antimicrobial substance reuterin, which has demonstrated efficacy in reducing gingivitis and associated gingival bleeding (Agossa et al., 2022; Garcia et al., 2022). Furthermore, after 4 weeks of probiotic consumption containing *L. reuteri*, it was observed a significant reduction in various oral pathogens, including *Aggregatibacter actinomycetemcomitans*, *Porphyromonas intermedia*, *Porphyromonas gingivalis*, *Treponema denticola*, and *Tannerella forsythia* (Jansen et al., 2021; Ochoa et al., 2023). Additionally, *L. reuteri* has been found to possess the capability to inhibit pro-inflammatory cytokines through the secretion of bacteriocins, reuterin, and reutericyclin. These compounds are recognized for preventing the growth of various pathogens, including bacteria, viruses, and fungi (Jang et al., 2022).

In a recent randomized clinical trial conducted by Volgenant and colleagues, the potential benefits of probiotic capsules containing one of two oral probiotic strains on gingival bleeding were investigated. Additionally, the authors assessed probiotics’ positive effects on oral health, including gingival health, dental plaque accumulation, and immunological and microbiological factors. Subjects were administered capsules containing *L. plantarum, and L. paracasei*. These findings underscore probiotic capsules’ potential to contribute to oral well-being by improving gum health, mitigating inflammation, and modulating the oral microbiome. Notably, the *L. paracasei* strain exhibited a more robust capacity to modulate the oral microbiome than the *L. plantarum* strain (Volgenant et al., 2022).

#### 5.3.3 Probiotics in the prevention of periodontal diseases

Periodontitis is a multifaceted disease with numerous causal factors involving microorganisms and the body’s response to them. The main etiology is the presence of biofilm bacteria on tooth surfaces. However, the disease’s evolution and severity depend on additional local factors, such as the accumulation of plaque and calculus, genetic influences, environmental factors, the individual’s overall health status, and lifestyle choices. Together, these factors collectively shape the development and progression of periodontitis (Sulijaya et al., 2019).

*Lactobacillus* sp., including *L. fermentum* and *L. gasseri*, can inhibit periodontal pathogens like *P. gingivalis*, *A. actinomycetemcomitans*, and *Prevotella intermedia* through the production of hydrogen peroxide, bacteriocrine antibacterial substances, and inorganic acids (Maitre et al., 2021; Mann et al., 2021). In the case of chronic periodontitis, *L. brevis* has shown promising anti-inflammatory effects due to its ability to significantly reduce key inflammatory markers such as prostaglandin E2 (PGE2) and matrix metalloproteinases (MMP). A recent study revealed that *L. brevis* can help prevent nitric oxide production, thereby reducing bacterial plaque accumulation (Hurtado-Camarena et al., 2023). In addition, regular consumption of *L. sali*var*ius* probiotics, taken three times a day for 8 weeks, has been associated with beneficial outcomes regarding periodontal health. Specifically, it has been linked to improvements in periodontal pocket probing depth and plaque index (Nedzi-Gora et al., 2020).

*B. lactis* administration in rats with periodontitis has been found to have several beneficial effects. These include a reduction in the levels of IL-1β and the regulation of the expression of TNF-α and IL-6. A recent study assessed the effects of *B. lactis* strain on periodontal parameters, highlighting its immunological and antibacterial properties by examining BD-3, TLR4, and CD4 levels in gingival tissues (Invernici et al., 2020). The composition of organic acids, such as lactic acid, produced by *Bifidobacterium* sp. can disrupt the outer membrane of Gram-negative bacteria. This disruption is known to reduce the adhesion of pathogens like *P. gingivalis* (Argandona Valdez et al., 2021). *Saccharomyces cerevisiae* is a yeast with probiotic properties due to beta-glucans in its cell wall. Beta-glucans can stimulate phagocytic activity and the production of inflammatory cytokines, thereby activating leukocytes (Nguyen et al., 2021). A study conducted by Garcia and collaborators aimed to evaluate the probiotic properties of beta-glucans. They found that this compound increased TGF-β1 concentration in patients with chronic periodontitis. This mechanism contributes to an anti-infective effect and enhances the potential for accelerated periodontal healing (Garcia et al., 2016).

Recent research has sparked interest in several newly discovered *Streptococcus* sp. due to their promising properties, including their ability to colonize oral environments, biocompatibility, and ease of experimental dosage determination. Recently, Deandra and collaborators reported that *S. sali*var*ius* strains possess the capability to inhibit the activity of periodontal pathogens such as *P. gingivalis*, *Prevotella intermedia*, *Fusobacterium nucleatum*, and *Aggregatibacter actinomycetemcomitans* (Deandra et al., 2023). Previous studies have indicated that *S. sali*var*ius* can help maintain immune homeostasis by targeting host cells by inhibiting the release of IL-6 and IL-8 triggered by *P. gingivalis*, *A. actinomycetemcomitans*, and *F. nucleatum* (Zhang Y. et al., 2022). In another research, individuals with stage II–III periodontitis who underwent mechanical periodontal treatment were monitored for periodontal outcomes. The study compared chlorhexidine-based toothpaste with toothpaste containing probiotics (including *Bifidobacterium* sp.) and chewing gum containing the same probiotics. After 3 months, an improvement was observed in both the inflammatory and microbial aspects of periodontitis (Butera et al., 2020). Salinas-Azuceno and collaborators conducted a study evaluating the potential of *L. reuteri* as monotherapy to improve periodontal health in a 30-year-old patient with periodontitis. The research indicated that *L. reuteri* exhibited a temporary, targeted antimicrobial effect, reducing the growth of specific harmful oral bacteria associated with periodontitis. During a month of probiotic consumption, the results revealed increased levels of beneficial oral bacteria, including *Actinomyces* sp., *Streptococcus* sp., *Gemella* sp., *Capnocytophaga* sp., and certain purple complex species (Salinas-Azuceno et al., 2022).

#### 5.3.4 Probiotics in halitosis

Halitosis, often called bad breath, can be caused by various factors related to oral and non-oral sources, including poor oral hygiene, periodontal diseases, oral tissue crusts, food impaction, unclean dentures, damaged dental restorations, oral cavity carcinomas, and respiratory and gastrointestinal infections. The key culprits behind halitosis are volatile sulfur compounds (VSCs), which can be categorized into oral and non-oral types depending on their source (Hampelska et al., 2020). Furthermore, an imbalance in the normal commensal microbiota can produce sulfur vapor components such as hydrogen sulfide, dimethyl sulfide, and methyl mercaptan (Yoo et al., 2019). Clinical trials have demonstrated the effectiveness of antimicrobial mouthwashes containing *S. salivarius K12* in reducing VSC-producing bacteria levels, which plays a crucial role in treating halitosis (Hyink et al., 2007). *S. salivarius* has been identified as a producer of salivaricin, a lantibiotic known to inhibit *S. pyogenes*, responsible for throat infections and oral malodor (Barbour et al., 2023). Another approach to combatting halitosis involves oral rinsing with a probiotic suspension of *Weissella cibaria*, which has been found to reduce malodor caused by *F. nucleatum* (Kang et al., 2006). *W. cibaria* produces hydrogen peroxide, which competes with secondary colonizers for attachment sites in the mouth. This competition reduces the reservoir of periodontal pathogens that produce volatile sulfur compounds and subgingival plaque (Kim et al., 2020).

In a randomized, double-blind, placebo-controlled trial, Lee and colleagues investigated the impact of oral probiotic *W. cibaria* tablets on halitosis. Participants took either *W. cibaria* or a placebo daily before bedtime for 8 weeks. The study revealed that for individuals with halitosis, the eight-week intake of this oral probiotic could serve as a beneficial intervention to reduce bad breath and enhance oral health-related quality of life (Lee et al., 2020). Patil and collaborators conducted a recent study to assess the effectiveness of *L. rhamnosus* in inhibiting bacteria responsible for halitosis. The research found that *L. rhamnosus* exhibited inhibitory effects on halitosis-causing bacteria such as *Tannerella forsythia* and *P. intermedia* after 48 h and on *P. gingivalis* after 72 h (Patil et al., 2023). In another recent randomized, double-blinded study, Han and colleagues aimed to explore the effects of tablets containing *W. cibaria* on halitosis. This study involved 100 adults with halitosis, randomly assigned to either the test group (*n* = 50) or the control group (*n* = 50). The researchers assessed improvements in bad breath, concentrations of VSCs, and the colonization of *W. cibaria*. The study found that the total VSC levels were significantly lower in the probiotics group. However, the difference between hydrogen sulfide and methyl mercaptan was insignificant, supporting the efficacy of *W. cibaria* in improving halitosis (Han et al., 2023).

### 5.4 Probiotics in the treatment of urogenital infections

Urogenital infections, including yeast vaginitis, bacterial vaginosis (BV), urinary tract infections (UTI), and non-sexually transmitted urogenital infections, constitute common reasons for women to seek gynecological care (Al-Badr and Al-Shaikh, 2013). The composition of normal vaginal commensals in healthy women can vary in premenopausal and postmenopausal stages. In healthy premenopausal women, Lactobacillus species typically predominate. These *Lactobacillus* species include *L. delbrueckii*, *L. brevis*, *L. crispatus*, *L. casei*, *L. jensenii*, *L. fermentum*, *L. plantarum*, *L. reuteri*, *L. salivarius*, *L. rhamnosus*, *L. gasseri*, and *L. vaginalis* (Petrova et al., 2015). Several factors contribute to these variations, including vaginal pH, glycogen levels, hormonal variations (especially estrogen fluctuations), and the menstrual cycle. Together, these factors affect the colonization and attachment of pathogens to vaginal epithelial cells. Elevated estrogen levels in healthy premenopausal women encourage the adherence of lactobacilli while discouraging the colonization of other pathogens. Conversely, in postmenopausal women, estrogen levels decrease, leading to changes in the vaginal microbiota that can contribute to urogenital infections (Witkin, 2015). Bacterial vaginosis (BV), the most prevalent urogenital infection, is primarily attributed to decreased *Lactobacillus* species and subsequent overgrowth of Gram-negative anaerobes (Mestrovic et al., 2021).

Probiotics offer a promising approach to restoring the balance of commensal organisms in the vagina, which can disrupt the growth of pathogenic organisms and inhibit biofilm formation (Barrea et al., 2023). Reid and collaborators initially reported the idea of repopulating the vagina with lactobacilli through oral probiotics (Reid et al., 2001). *Lactobacillus* sp. combat vaginal pathogens through various mechanisms, including the production of antimicrobial agents like bacteriocins (Gaspar et al., 2018) and biosurfactants that alter the surface tension of the environment, preventing pathogen adhesion and further inhibiting their spread in the bladder. Lactobacilli also play a crucial role in maintaining vaginal pH (Ballini et al., 2018). Research has shown that administering the probiotic *Lactobacillus rhamnosus* GR-1 to premenopausal women can increase the expression levels of antimicrobial defenses (Barrea et al., 2023). A randomized, double-blind, placebo-controlled pilot study included 81 premenopausal adult women who experienced recurrent UTIs. The study aimed to evaluate a product containing two Lactobacilli strains and cranberry extract to prevent recurrent UTIs in premenopausal women. The results demonstrated that this product was safe and effective in preventing recurrent UTIs in this group (Koradia et al., 2019). Another study led by Armstrong and collaborators, conducted as part of a phase 2b trial, investigated the impact of the LACTIN-V product based on *L. crispatus* on genital immunology and vaginal microbiota. The findings from this study revealed the role of LACTIN-V in reducing genital inflammation, serving as a biomarker of epithelial integrity (Armstrong et al., 2022).

More recently, Ansari and collaborators investigated the benefits of *Lactobacillus* sp. probiotics in improving vaginal dysbiosis and facilitating the colonization in asymptomatic women. This study included 36 patients who received a combination of *L. acidophilus*, *L. rhamnosus*, and *L. reuteri* and revealed that oral administration of these probiotics improved vaginal dysbiosis in asymptomatic women (Ansari et al., 2023). A recent randomized, double-blind trial (ISRCTN34840624, BioMed Central) conceptualized by Mandar and collaborators investigated the impact of the vaginal probiotic capsules containing *L. crispatus* strains in 182 patients with BV and vulvovaginal candidiasis (VVC). The study highlighted the benefits of vaginal capsules in improving the signs, symptoms, and amount of discharge and itching/irritation in BV and VVC patients (Mandar et al., 2023). Another clinical trial (KCT0005881, Clinical Research Information Service, the Republic of Korea) was conducted to evaluate the effectiveness of a complex of five strains of probiotic candidates in restoring vaginal health in 76 reproductive-aged women. The consequent analyses confirmed the increment of *L. plantarum*, concomitant with inhibiting harmful bacteria such as *Mobiluncus* spp., *Gardnerella vaginalis*, and *Atopobium vaginae*. This clinical trial demonstrated that this probiotic complex can be used for treating BV, as it improves the vaginal microbiota (Park et al., 2023).

Preterm birth (PTB) is a significant global challenge and ranks second leading cause of neonatal mortality (Keelan and Newnham, 2017). Research has highlighted a robust connection between BV and PTB. Consumption of *Lactobacillus* sp. during both early and late pregnancy has been shown to reduce the incidence of PTB-related infections, inflammation, and even conditions like pre-eclampsia (Zheng et al., 2019). A notable characteristic of healthy female reproductive tracts is the predominance of a less diverse community of *Lactobacillus* sp. (Madhivanan et al., 2015). In contrast, a highly diverse vaginal microbiome is associated with BV and serves as a primary risk factor for PTB and the acquisition of pelvic inflammatory disease (Holdcroft et al., 2023).

### 5.5 Probiotics in cancer prevention and treatment

Probiotics have emerged as promising agents in the fight against cancer, showing their potential in laboratory experiments (*in vitro*) and animal studies (*in vivo*). Various probiotic strains have exhibited their ability to combat different types of cancer. *Lactobacillus* strains (*L. paracasei* SR4, *L. casei* SR1, and *L. casei* SR2) have demonstrated anticancer effects against cervical cancer cells (HeLa) by increasing the expression of apoptotic genes like BAX, BAD, caspase-8, caspase-3, and caspase-9 while reducing the activity of the anti-apoptotic BCl-2 gene (Riaz Rajoka et al., 2018). Studies have revealed that a compound produced by *Enterococcus thailandicus* possesses significant anticancer properties, particularly against liver cancer cells (HepG2). Yogurt fortified with probiotics has demonstrated the ability to hinder the development of colon tumors induced by 1,2-dimethylhydrazine in laboratory mice (BALB/c mice), suggesting its potential as an anticancer dietary option. An aqueous extract of *Bifidobacterium* sp. has been shown to induce apoptosis in non-small cell lung cancer cells, effectively inhibiting the invasive behavior of cancer cells (Ahn et al., 2020). Various mixtures of probiotics, including *L. casei-01*, combined with dairy beverages, have demonstrated antiproliferative and apoptotic effects on human prostate cell lines (Rosa et al., 2020).

*Bacillus Calmette-Guerin* (BCG) has long been employed as preventive immunotherapy for recurrent superficial bladder cancer (Guallar-Garrido and Julián, 2020). Additionally, regular lactic acid bacteria consumption is considered a preventative measure against bladder cancer (Andolfi et al., 2020). Probiotics enhance the expression of junctional molecules, thereby preserving the integrity of the intestinal barrier. They also contribute to the production of IgA and short-chain fatty acids, which impede the adherence and proliferation of pathogens (Engevik and Versalovic, 2017).

Moreover, *L. plantarum* releases polysaccharides with recognized antitumor properties. These polysaccharides act by downregulating the expression of MAPK mRNA and upregulating PTEN (Asoudeh-Fard et al., 2017). Research on *L. lactis* strains has revealed their capacity to secrete IL-10 and INF-β, as well as express antioxidants that mitigate the generation of reactive oxygen species (ROS) and reduce colonic damage in animal models (Zhao et al., 2023).

A recent randomized clinical trial conducted by Liu and colleagues confirmed that the perioperative use of probiotic supplements can reduce postoperative infections, improve short-term clinical outcomes, and alleviate common inflammatory symptoms in patients with gastric cancer receiving neoadjuvant chemotherapy (Liu G. et al., 2022; Liu Y. et al., 2022).

The incidence of oral cancer is closely linked to changes in the oral and intestinal microbiota. Variations in the levels of essential vitamins and nutrients can trigger the production of inflammatory cytokines, leading to various pathological conditions (Kany et al., 2019). Certain microbes, including *F. nucleatum*, *P. gingivalis*, and *P. intermedia*, have strong associations with the initiation and progression of oral cancer (Atanasova and Yilmaz, 2014). Among the microbial species extensively studied in this context are *Actinomyces* sp., *Clostridium* sp.*, Enterobacteriaceae* sp., *Fusobacterium* sp., *Haemophilus* sp., *Prevotella* sp., *Veillonella* sp., and *Streptococcus* sp. These microorganisms exhibit a robust correlation with precancerous oral lesions and the development of oral cancer (Hu et al., 2016).

Inflammation of the oral mucosa is a frequently encountered condition known as oral mucositis (OM). OM presents with symptoms such as redness (erythema), ulceration, pain, difficulty swallowing (dysphagia), and nutritional deficiencies. This condition is a common consequence in individuals undergoing radiotherapy and chemotherapy. It is primarily attributed to several factors, including the detrimental effects of reactive oxygen species leading to the death of basal stem cells, DNA damage, and inflammatory cytokines synthesis (Lalla et al., 2014).

*L. rhamnosus GG* (LGG) is a naturally occurring gut commensal bacterium known for its anti-inflammatory properties and has been a pioneer in oncology research (Banna et al., 2017). LGG maintains the equilibrium of the intestinal mucosa by neutralizing harmful pathogens and toxins, effectively preventing breaches in the mucosal barrier through a high-affinity binding system (Okumura and Takeda, 2018). One of its key mechanisms involves regulating the production of IgA (Wang et al., 2017). LGG is also recognized for producing increased levels of geniposide, an anticancer molecule, and its potential as a beneficial adjuvant during cancer treatment. Studies have indicated that LGG, through its probiotic action, can protect epithelial stem cells from radiation-induced damage and reduce their apoptosis. It modulates cyclooxygenase-2 and stimulates the release of prostaglandin E2 (PGE2) (Desai et al., 2018). Furthermore, LGG plays a role in immune system modulation by secreting various cytokines, including IL-6, IL-10, IL-1β, TNF-α, IL-12, and p40. These actions help reduce inflammation, regulate epithelial function, and maintain intestinal mucosa integrity (Andrews et al., 2018). In the context of cancer treatment, *L. brevis CD2* lozenges have been found to reduce the occurrence of oral mucositis in patients undergoing high-dose chemotherapy (Sharma et al., 2017). Additionally, *L. brevis* lozenges have shown benefits in reducing oral ulcers in individuals with recurrent aphthous stomatitis (Abruzzo et al., 2020). These findings underscore the potential of probiotics such as LGG and *L. brevis* in alleviating oral mucosal problems and promoting overall health.

Recently, a meta-analysis conducted by Frey-Furtado and colleagues examined nine articles to evaluate the therapeutic effectiveness of probiotics in managing oral mucositis. Among these studies, four clinical trials reported a decrease in the severity of oral mucositis by using specific strains of bacteria, including *Lactobacillus* (*L. casei* and *L. brevis* CD2) and *B. clausii* UBBC07. Preclinical studies revealed the positive effects of *L. lactis*, *L. reuteri*, and *S. salivarius K12* in reducing the severity of oral mucositis and the size of ulcers (Frey-Furtado et al., 2023). Moreover, two distinct meta-analyses conducted by research teams from Taiwan and China, encompassing a total of 19 randomized clinical trials, investigated the potential of probiotics in preventing oral mucositis induced by cancer therapy and in managing the occurrence of chemotherapy-induced diarrhea and oral mucositis. Both studies revealed the effectiveness of probiotics in preventing and alleviating cancer therapy-induced oral mucositis and addressing adverse reactions associated with chemotherapy (Feng et al., 2022; Liu G. et al., 2022; Liu Y. et al., 2022).

### 5.6 Probiotics in the treatment of anemia

Numerous studies have highlighted a significant connection between gut microbiota and iron deficiency. Furthermore, research has illuminated the reciprocal relationship between iron deficiency and the composition of gut microbiota, as well as the beneficial impact of probiotic bacteria on enhancing iron absorption (Sandberg et al., 2018; Hadadi et al., 2021).

Folic acid, a water-soluble B vitamin crucial for addressing and managing anemia, is derived from probiotic bacteria, including *L. lactis*, *L. cremoris*, *B. pseudocatenulatum*, *Candida famata*, *B. adolescentis*, *Candida glabrata*, *Candida guilliermondii*, *S. cerevisiae*, *Yarrowia lipolytica*, and *Pichia glucozyma*. These bacteria are harnessed to enhance the intestinal absorption of folic acids. In addition, *P. denitrificans* and *P. shermanii* have found utility in treating vitamin B12 deficiency. Lactic acid-fermented foods are instrumental in increasing iron absorption and are employed in managing anemic patients. They help optimize the pH of the digestive tract and activate the enzyme phytase, which plays a role in nutrient absorption. Furthermore, a combination of probiotic bacteria has been incorporated into food to treat megaloblastic anemia. These probiotics promote colonic fermentation and counteract the adverse effects of antibiotics (Ohtani et al., 2014).

Numerous studies have shed light on the positive effects of probiotics, particularly *Lactiplantibacillus plantarum 299v*, in addressing iron deficiency anemia and enhancing non-heme iron absorption. A study among Caucasian Europeans revealed that *L. plantarum 299v* had a beneficial impact in preventing iron deficiency anemia and improving the absorption of non-heme iron (Vonderheid et al., 2019). Korcok and collaborators investigated the role of *L. plantarum 299v*, sucrosomal iron, and vitamin C in preventing and treating iron deficiency, administering vitamin C and iron to one group. In contrast, the second group received additional *L. plantarum*. The results showed that the second group had higher iron blood levels due to increased iron absorption, highlighting the additive effect of *L. plantarum 299v* (Korcok et al., 2018). Adiki and colleagues demonstrated that using *L. plantarum 299v* with pear millet enhanced iron absorption. Interestingly, they noted that the increased dose of probiotics did not directly influence hemoglobin and hematocrit levels (Adiki et al., 2019). In individuals with an increased need for iron supplementation, Hoppe and colleagues reported that adding *L. plantarum 299v* to a meal improved iron bioavailability (Hoppe et al., 2017). In a double-blind clinical trial involving patients undergoing hemodialysis, probiotic supplementation containing *L. acidophilus*, *B. bifidum*, *B. lactis*, and *B. longum* led to a significant increase in serum hemoglobin levels compared to the placebo group (Haghighat et al., 2021). A recent randomized placebo-controlled study among athletes found that daily high-protein concentrated pro-yogurt enriched with whey protein isolates significantly increased hemoglobin levels. The study suggested that probiotics in concentrated yogurt, alongside enhanced iron bioavailability through mineral binding, can positively influence iron status and help improve athletic anemia and performance (Gomaa et al., 2022).

### 5.7 Probiotics in the treatment and prevention of obesity

Obesity is a significant risk factor for various health conditions, including hypertension, coronary heart disease, and type II diabetes. Multiple factors contribute to obesity, including dietary choices, physical activity levels, age, genetics, and developmental stage (Leon Aguilera et al., 2022). In recent times, a novel approach to managing and reducing obesity has emerged through the use of probiotic bacteria. Probiotics have demonstrated their potential to reduce weight gain and combat obesity effectively, regulating food intake and promoting prolonged feelings of satiety, reducing fat deposition in the body, enhancing energy metabolism, and increasing insulin sensitivity (Daniali et al., 2020).

Research into the use of probiotics for managing obesity has yielded several significant findings from various studies. A comprehensive meta-analysis of 105 trials involving 6,826 obese patients concluded that probiotics moderately improved body mass index (BMI) and weight, resulting in a 3–5% reduction. However, there was no statistically significant impact on parameters such as glycated hemoglobin, cholesterol, triglycerides, insulin resistance, or liver function. Notably, in overweight (but not obese) individuals, probiotics, especially strains like bifidobacteria (*B. breve*, *B. longum*), *S. salivarius subsp. thermophilus*, and lactobacilli (*L. acidophilus*, *L. casei*, *L. delbrueckii*), brought about improvements in waist circumference, body fat mass, visceral adipose tissue mass, BMI, and body weight (Koutnikova et al., 2019). In another meta-analysis focusing on women with polycystic ovary syndrome (PCOS), probiotic supplementation was found to have a positive impact. It improved BMI, weight, insulin levels, triglycerides, and low-density lipoprotein (VLDL) cholesterol levels. However, it did not significantly affect dehydroepiandrosterone sulfate levels or total LDL and HDL cholesterol levels (Tabrizi et al., 2022). A comprehensive review encompassing thirty-three randomized clinical trials involving individuals with overweight and obesity revealed that approximately 30% of the trials reported reductions in body weight and BMI, while 50% showed significant reductions in waist circumference and total fat mass (Guedes et al., 2023). In a PROSPERO meta-analysis comprising eleven randomized controlled trials involving morbidly obese patients undergoing bariatric surgery, probiotics demonstrated beneficial effects. The pooled analysis indicated that probiotics could help regulate triglycerides, reduce weight, and influence dietary intake, particularly carbohydrates and fiber. The findings suggested that probiotics might delay the progression of liver function impairment, improve lipid metabolism, and reduce weight and food intake (Wang et al., 2023b).

Moreover, the microbiota administration from a healthy individual to an obese person can alter the composition of the recipient’s microbial flora. Recent studies have revealed that probiotics play a role in reducing serum cholesterol levels through mechanisms involving the production of short-chain fatty acids and the conjugation of bile salts (Jia et al., 2023; Momin et al., 2023).

### 5.8 Probiotics in COVID-19

The global pandemic caused by coronavirus disease 2019 (COVID-19) has tragically impacted countless lives. Despite the widespread adoption of rigorous public health measures such as social distancing, personal care, mask mandates, and lockdowns, the virus continues to proliferate, escalating infections and fatalities worldwide. The primary culprit behind this disease is severe acute respiratory syndrome coronavirus 2 (SARS-CoV-2), which distinguishes itself from previously identified coronaviruses. Consequently, existing antiviral treatments have demonstrated limited effectiveness (Singh and Rao, 2021). In this context, natural products like probiotics and their derivatives are being investigated for their potential contributions to pandemic prevention, treatment, and overall management, extending to post-pandemic periods and other severe infections (Brahma et al., 2022; Banerjee et al., 2023).

Currently, no direct evidence suggests that probiotics can directly inhibit coronaviruses. However, research has shown that certain probiotic strains have inhibitory effects on various viruses, as reviewed by multiple researchers (Olaimat et al., 2020; Tiwari et al., 2020; Singh and Rao, 2021). For example, in an animal study, *L. plantarum* was found to effectively reduce the proliferation of the influenza A (IFV) virus in the lungs in a dose-dependent manner. This probiotic also strengthened the Th1 immune response and increased the production of secretory IgA, contributing to the elimination of IFV from the lungs (Kawashima et al., 2011). This suggests that probiotic consumption can help reduce viral loads and enhance immune responses, which is particularly important during the pandemic. Bacteriocidins produced by *S. salivarius* 24 SMB have been shown to inhibit the common respiratory pathogen *S. pneumoniae* (Santagati et al., 2012). Additionally, *L. rhamnosus*, alone or in combination with *B. animalis subsp. lactis* Bb-12 has been found to reduce upper respiratory infections in children. It modulates the immune system and reduces nasal colonization with *S. aureus* and *S. pneumoniae* in adults. *S. sali*var*ius* K12 has effectively reduced the incidence of infectious episodes in pharynx-tonsillar infections caused by group A beta-hemolytic streptococci and in children with otitis media. It has also been shown to prevent the recurrence of tonsillitis and pharyngitis (Di Pierro et al., 2016).

While limited data is available, specific probiotic strains such as *L. gasseri* SBT2055, *L. rhamnosus* CRL1505, *B. bifidum*, and *B. subtilis* warrant further exploration for their potential roles in managing COVID-19. Moreover, probiotic bacteria exhibit antioxidant capabilities, which are especially pertinent for COVID-19 management due to the critical role of redox homeostasis in curbing disease progression (Starosila et al., 2017; Singh and Rao, 2021).

**Figure 4:**
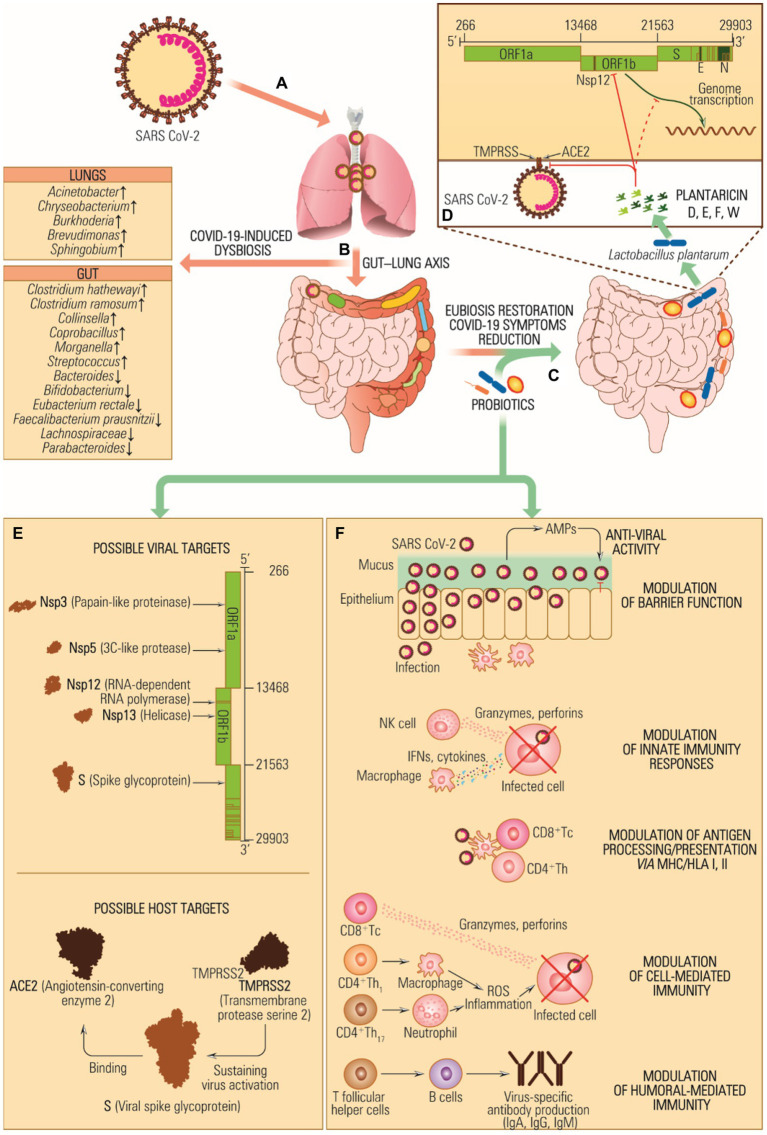
Effects of probiotics on SARS-CoV-2 infection. **(A)** The virus enters the body, infecting the respiratory system, including the lungs. **(B)** Via the gut-lung axis, SARS-CoV-2 virus induces dysbiosis in the lungs and colon. **(C)** Dietary supplementation with probiotics is effective in restoring eubiosis and relieving symptoms produced by SARS-CoV-2 infection. **(D)** Example of beneficial effect produced by the probiotic *Lactobacillus plantarum*, whose plantaricins D, E, F, and W inhibit the binding of viral spicular glycoprotein S (S1 subunit) to the cellular ACE2 receptor, they also and binding and inactivate RNA-dependent RNA polymerase (Nsp12), preventing transcription of the viral genome. **(E)** Different compounds secreted by probiotics may target some viral proteins, including Nsp3, Nsp5, Nsp12, Nsp13 and S, or some host cell-expressed proteins, ACE2, which serves as a receptor for viral attachment, and TMPRSS2, which facilitates activation of the viral glycoprotein S, with activation of the virus and its cell binding. **(F)** Probiotics participate in the modulation of barrier function and antiviral activity of epithelia, modulate innate immune response, antigen processing and presentation by antigen-presenting cells via MHC or HLA I and II molecules, and cellular and humoral mediated immunity.

Similar to other coronaviruses, SARS-CoV-2 employs the angiotensin-converting enzyme 2 (ACE2) for cellular entry and relies on the transmembrane protease serine 2 precursor (TMPRSS2) for priming and replication within the host (Ahlawat et al., 2020; Hoffmann et al., 2020)(Figure 4). This has suggested that ACE inhibitors could have a role in COVID-19 management (Meng et al., 2020). Notably, certain probiotics, especially lactic acid bacteria, are known to produce bioactive peptides that function as ACE inhibitors (Kim et al., 2021; Bhattacharya et al., 2022). Additionally, the lung-gut axis and its interplay with microbiota, in conjunction with probiotics, have been proposed for investigating COVID-19 management, building on prior work by Ahlawat et al. (2020).

Hence, there is a need to delve into the potential of probiotics and their derivatives, individually and in combinations, which exhibit promising activities that could be harnessed in the future (Singh and Rao, 2021).

Two recent meta-analyses suggest that probiotics may positively impact the overall symptoms experienced by individuals with COVID-19. Specifically, the results showed that probiotics have the potential to restore equilibrium in the intestinal microbiota, decrease the duration of diarrhea, enhance respiratory symptoms such as cough and dyspnea, and reduce the length of hospitalization (Tian et al., 2023; Zhu et al., 2023).

The potential application of microbiome or probiotic therapy holds promise as an emerging therapeutic approach due to its foundation on the multifactorial mechanism of action exhibited by beneficial bacteria in combating respiratory viral diseases (De Boeck et al., 2022). This adjuvant is characterized by its immunomodulatory properties, including the capacity to stimulate interferon production, mitigate excessive immune responses, and inhibit cytokine storms, thereby preventing pathological inflammatory states through immune response modulation (Nayebi et al., 2021). Other studies on different probiotic formulas investigated a throat spray containing specific antiviral lactobacilli. These studies suggest that this formula can decrease nasopharynx viral loads and alleviate acute symptoms (De Boeck et al., 2022). The administration of oral probiotic formula based on *Lactiplantibacillus plantarum* strains and *Pediococcus acidilactici* shows a reduced nasopharyngeal viral load, lung infiltrates, and digestive and non-digestive symptoms duration. The observed effect of the therapy resulted in an elevation of specific IgM and IgG antibodies targeting SARS-CoV2. This finding implies that the intervention may predominantly influence the gut-lung axis through communication with the host’s immune system rather than altering the colonic microbiota (Gutierrez-Castrellon et al., 2022). However, studies based on the microbiome analysis confirmed that the abundance of *Lactobacillus rhamnosus* was notably higher in individuals who were administered probiotics with *L. rhamnosus* GG strain compared to those who received a placebo (Tang et al., 2021; Wischmeyer et al., 2022).

Age-related immune response modifications make the elderly substantially more susceptible to COVID-19. Vaccination is crucial in mitigating the severity and fatality rates among older adults. However, it is important to note that age-related deficiencies in immune response might result in diminished levels of protection following vaccination when compared to younger cohorts. Probiotic supplements have been recognized as a supplementary measure to enhance immune function and improve the particular immune responses to vaccines in the aged demographic. The administration of *Loigolactobacillus coryniformis* K8 has shown promising effects, such as increasing the IgA response in correlation with the activation of TGF-β, a key signaling molecule involved in immune regulation and tolerance. Furthermore, the positive immune response induced by K8, as evidenced by the enhanced response to the BNT162b2 mRNA COVID-19 vaccine, suggests that individuals who receive this probiotic may have a more robust immune response if they are exposed to the live virus in the case of infection. This finding underscores the potential benefits of probiotics like K8 in strengthening the body’s defenses against viral threats, including COVID-19 (Fernández-Ferreiro et al., 2022).

## 6 Conclusions and future perspectives

The application of microbial therapy, particularly probiotic bacterial species, has garnered increasing attention in recent years. Researchers have been diligently working to provide conclusive evidence regarding the beneficial effects of probiotic strains on human health and disease outcomes (Tiwari et al., 2020). Probiotic bacteria offer many advantages, including their anti-inflammatory, anti-cancer, antimicrobial, antioxidant, and immunomodulatory properties. These properties enable them to play crucial roles in shaping the physiological and metabolic functions of the host while also combatting pathogenic microbes. The potential contributions of probiotics to the prevention and treatment of conditions such as diabetes, obesity, and cancer have opened up exciting and rapidly growing areas of research (Leong et al., 2018). Incorporating probiotics into one’s diet through dairy products and fermented foods represents a straightforward and cost-effective means of enhancing human health. As research advances, there is a growing emphasis on evaluating new strains of human gut microbes and exploring their potential applications in biomedical and clinical research. This ongoing exploration promises to uncover new directions and opportunities for harnessing the benefits of probiotics for human well-being (Nataraj et al., 2020).

Selecting the appropriate probiotic products can be challenging, as their effectiveness hinges on several factors, including the specific strain, the type of disease or condition being targeted, and the appropriate dosage. Probiotics operate through various mechanisms to combat different pathogens, and what works for one disease may not necessarily be effective for another. For example, while LGG effectively prevents antibiotic-associated pediatric diarrhea, it may not yield the same results for diseases like Crohn’s disease, CDI, nosocomial infections, or traveler’s diarrhea (Li et al., 2019). Standardizing the required dosage of probiotics is a complex task. Achieving the optimal number of viable probiotic cells for effective gut colonization depends on several factors, including the manufacturing processes, quality control measures, interactions between different bacterial species administered together, and the ability of probiotics to withstand the acidic and bile-rich environment of the digestive system. It’s also important to note that, like antibiotics, there is a potential risk of horizontal transfer of AMR-related genes from probiotic strains to other co-infecting pathogens and vice versa. This underscores the need for careful consideration and monitoring when using probiotics, especially when antimicrobial resistance is a concern (Silva et al., 2020).

Despite their potential health benefits, the clinical application of probiotics is facing several challenges that warrant careful consideration and adherence to safety protocols. Probiotics can face viability problems during storage, particularly at room temperature. Ensuring the stability and shelf-life of probiotic products is essential to maintain their effectiveness (Khan A. et al., 2023; Khan M. T. et al., 2023). Probiotics may vary in their ability to colonize the intestinal tract and tolerate the conditions within the gut. There is a concern that probiotics could acquire virulence genes from opportunistic or pathogenic organisms, potentially compromising their safety. Like other bacteria, probiotics can transfer antibiotic-resistance genes within the GIT, raising concerns about the spread of antimicrobial resistance. Some probiotic strains, such as Bacillus species, can produce heat-stable toxins like amylosin, which can potentially induce food poisoning. In light of these challenges, healthcare professionals and researchers must exercise caution when incorporating probiotics into clinical applications (Yadav et al., 2022). It is essential to thoroughly evaluate probiotic strains, their safety profiles, and their specific intended uses. Additionally, adherence to safety protocols and rigorous monitoring can help mitigate potential risks associated with probiotic administration (including storage conditions, gene transfer, and toxin production) while maximizing their potential health benefits (Siciliano et al., 2021).

Developing clinically efficacious probiotics remains challenging and must adhere to various requirements to ensure their safety and effectiveness. Several key considerations in developing and evaluating probiotics include determining the minimum effective dose, including adequate controls, and selecting and combining probiotics relevant to the targeted health outcomes (Brahma et al., 2022). Probiotics hold promise for reducing antibiotic use and addressing various health issues. However, concerns are related to the over-the-counter availability of numerous probiotic combinations with questionable efficacy and the potential for irrational use and undefined dosing. Therefore, researchers and healthcare professionals must exercise caution and conduct rigorous evaluations when considering probiotics for clinical applications (Yadav et al., 2022).

Microbiome therapy aims to establish a healthy native microbial environment in the gut to prevent dysregulation and promote survival. This therapeutic approach leverages the potential of microbes to restore imbalances and enhance the host’s survival by influencing various metabolic, nutritional, and physiological pathways, particularly those that affect pathogens. The effectiveness of microbiome therapy has proven challenging under diverse circumstances. Furthermore, research in this field has predominantly relied on rodent models, necessitating a shift toward more human-focused studies. Ensuring the success of microbiome therapy is crucial to the stability and resilience of clinically relevant microbial strains.

In summary, advancing our understanding of these innovative therapeutic strategies is of paramount importance. Until these approaches are well-defined, structured, and readily available in the market, it remains imperative to establish standards for controlling conventional chemical antibiotics, which can significantly disrupt human and animal microbiota in daily consumed foods. Addressing various safety and regulatory concerns is essential for the successful execution of clinical trials involving microbiome therapy. A regulatory framework must be crafted to ensure the biosafety of these therapies, minimizing adverse effects and the inadvertent release of genetically modified microbes into the environment. Additionally, evaluating modified probiotics’ long-term safety and efficacy is vital for their sustained therapeutic benefits.

## Author contributions

O-AP: Writing – original draft, Writing – review & editing. ICB: Writing – review & editing. A-GN: Writing – review & editing. MC: Writing – review & editing. GAG: Writing – original draft, Writing – review & editing. R-EC: Writing – review & editing. GM: Writing – review & editing. COV: Conceptualization, Writing – original draft, Writing – review & editing.
